# Illinois State Medical Society

**Published:** 1880-06

**Authors:** 


### Society Reports.

### ARTICLE V.

ILLINOIS STATE MEDICAL SOCIETY. Annual Meeting at Belleville, Ill., May 18, 19 and 20, 1880.

FIRST DAY. MORNING SESSION.

In the temporary absence of the President, the First Vice President, Dr. G. W. Jones, of Danville, occupied the chair. Prayer was offered by Rev. Mr. Van Treese. An address of welcome in behalf of the local Committee of Arrangements was made by Dr. W. West, Chairman.

The address of welcome on behalf of the citizens, by Judge Snyder, was postponed to the evening. Various matters of routine business were attended to, when the President, Dr. E. Ingals, of Chicago, arrived and occupied the chair. A letter of resignation of the office of Permanent Secretary was received from Dr. N. S. Davis. Dr. Davis gave as a reason for his resignation ill health, and expressed regret at being obliged to take this step after a service of thirty years.

His resignation was accepted and a committee appointed to prepare resolutions that should properly express the sentiments and regrets of the Society at this step.

AFTERNOON SESSION.

Dr. E. Ingals read his address as President. It dealt chiefly with the hygiene of cities, and was especially applicable to Chicago.

First were considered the duties of the government and of the profession in sanitary matters. He assumed that much of the disease we suffer might be avoided by drinking pure water and breathing pure air, and this led to a consideration of the sewerage of Chicago and its water supply. He advocated the enlarging of the canal to the capacity of a river; the use of large quantities of water in our houses and sewers; the ventilation of sewers in the streets, and of house-drains by pipes reaching above the house, and of the house itself by heating with open grate fires, being careful that the air supply is pure, and especially that it be not drawn from beneath the house. Also, that government supervise the sewerage, plumbing and air supply of buildings—private as well as public. He claimed there was no water superior to that of the Northern Lakes, and predicted that Illinois would become populous and wealthy enough to supply this to its interior cities for domestic use. He advocated, so far as possible, the isolation of the sick from the well, and official surveillance of highly infectious and dangerous diseases, as smallpox, scarlet fever, yellow fever and Asiatic cholera; the use of disinfectants and the placing of warning cards. Also, that government should prevent ignorant people and charlatans from practicing the healing art, and prescribe the attainment necessary to enable any person to receive the degree of Doctor of Medicine. He advised that authors of papers presented to the Society be allowed to contribute them to medical journals either before or after their publication in the transactions.

Dr. Crummer, of Warren, then read the main report on

*Practice of Medicine.*—It embodied a discussion of diphtheria, with an account of an epidemic of this disease during the past fall and winter.

He discussed the evidence bearing on the contagiousness of diphtheria, and added to this evidence in the affirmative from his experience. Diphtheria was brought into one small community (having fifty-nine school children) by two boys who had visited a neighborhood at some distance where diphtheria was prevalent. In a few weeks the disease spread to fifty-eight people, with a mortality of thirty per cent.

He advocated the prompt isolation of all people sick with diphtheria. It was criminal almost to allow the uninfected children to remain in the same house with the infected. In one case he had had constructed a wood shanty in which to isolate the well children of a family where a case of diphtheria had occurred.

In the epidemic which he reported, two cases out of seventeen cases of severe nasal involvement had recovered.

His local treatment of the throat consisted in, among other things, the application of solutions of tannic acid. For the nasal cavities when they were involved he used a four per cent. solution of carbolic acid, or a solution of salicylic acid of the same strength, injected freely into the nose.

He spoke next of an epidemic of

*Pneumonia* which had visited Northern Illinois during the past winter. It had been quite widespread—the number of cases being greater than for years previously—but the mortality was low.

An extensive correspondence with many members of the profession in relation to this disease had shown that the practice as to the use of blisters and veratrum viride was very variable. There was quite a unanimity in the use of considerable doses of alcoholic stimulants for all cases that assumed a form of great depression.

Dr. F. B. Haller, of Vandalia, read a supplemental report on Practical Medicine, which consisted in an account of an epidemic of

*Mumps* which had occurred in his neighborhood during the past winter season. Nothing so widespread had ever occurred in his experience. Whole communities were attacked with this disease. Schools were, for a time, suspended for want of pupils. The cases were of all varieties in severity, but the proportion of severe cases was large. Metastasis was common. It occurred most in males, especially boys. A few cases occurred among grown women, in which the parts involved were generally the mammary glands, very rarely the ovaries. Girls escaped these complications. In a few cases inflammation of the meninges of the brain took place as a complication, and in some the symptoms were quite alarming. The temperature was as high as 105° F., the tongue dry; delirium was present and a typhoid state, yet no deaths resulted.

The complicating inflammation of the testicles was found most in the severer cases of parotitis.

The epidemic had witnessed no fatal cases, and no cases of damage of testicle had so far been noted among the victims of this visitation.

The treatment was expectant and palliative, poultices and Dover's powder forming a large part of it.

Dr. Bird, of Quincy, in discussing Dr. Crummer's report on diphtheria, advocated the Billington treatment, and had not thought isolation much of a necessity.

Dr. Hollister hoped for a better study and more evidence as to the bacterial character of the poison of diphtheria. He advocated the application to the diphtheritic patches of the liquid sub-sulphate of iron.

Dr. C. T. Parkes advocated the combination of a vigorous tonic treatment with a vigorous local one. For the latter purpose he would use the liquor ferri persulphates and glycerine in equal parts for application to the throat, and for the nose, when involved, solutions of boracic acid, to be freely used by injection.

Dr. W. Hill, of Bloomington, read the chief report on

*Surgery.*—He discussed the whole range of the recent important improvements in modern surgery, but paid special attention to antiseptic surgery, extirpation of floating kidney, extirpation of cancers of the rectum and treatment of rodent ulcer.

He recommended thymol as being equal to carbolic acid for the spray in the Lister treatment.

He quoted several records of cases of extirpation of the rectum. The operation seemed to be a feasible one. He had witnessed one operation of this kind in Cook County Hospital by Dr. Isham.

He advocated the treatment of rodent ulcer by the scraping-out process, and related three cases of successful operation by this method, the disease not having returned in either case. He did the scraping with the handle of a scalpel, and made it very thorough. Every particle of diseased tissue, down to perfectly healthy structure, was to be removed in this way.

He read a supplemental report by Dr. A. C. Rankin on

*Conservative Surgery*, in which were detailed some cases of very severe crushing injuries of the extremities, which recovered with useful limbs under conservative management.

Dr. J. G. Harvey read a report of two cases, one of extirpation of the parotid gland, the other an unusual case of hernia in an old woman.

In the latter case there was a band of fibrous tissue constricting the knuckle of gut inside the abdominal wall, making a strangulation that was not relieved by reduction of the hernia within the sac.

Dr. Bird, in discussing these reports, advised the attempt, in extirpating tumors of the rectum, to save the sphincter ani muscle. He had once done this successfully. He used the electro-cautery instead of the knife.

Dr. C. T. Parkes gave his experience and observation, in Cook County Hospital and elsewhere, with thymol in the antiseptic spray. It was not as valuable as carbolic acid. By Callander's method of dressing without spray, but using a two and one-half per cent. watery solution for freely washing wounds and wounded surfaces, or an eight per cent. solution in oil for the same purpose, the results were as good as by the Lister process with thymol.

Rectotomy was a very hazardous operation, the mortality of which was so large that it ought never to be done except for a very small epithelioma. Whenever the carcinomataous infiltration involved two or more inches of the intestine and encircled the canal it was useless to operate, if the teachings of experience were of any value. He had witnessed five operations, with four deaths; in three of these the peritoneal cavity had been opened. This accident was likely to happen whenever, during the operation, the tumor was drawn forcibly downward. This latter maneuver ought never to be made, nor should stitches be inserted into the cut end of the gut.

Dr. Hill spoke of his method of treating strangulated hernia. It was by carrying his finger or thumb up into the ring, forcing the skin before it, and dilating the ring by pressure upward until reduction without a cutting operation was possible.

Since he had used this method he thought he had operated on one hundred and fifty cases with success, and without encountering a case which this procedure would not relieve.

SECOND DAY. MORNING SESSION.

Dr. G. W. Nesbitt, of Sycamore, as Chairman of the Committee on Obstetrics, read his report. His effort was chiefly devoted to a consideration of the necessity that beginners in the practice of medicine have practical instruction in obstetrics. He thought this was the direction in which medical colleges could best improve their systems of instruction. In no department were young graduates so defective as in this. He advocated lying-in-hospitals in connection with medical colleges, and said that on the so-called cottage plan the expense need not be great.

Dr. W. T. Montgomery, Chairman of the Committee on Ophthalmology and Otology, read an interesting paper on

*Suppurative Inflammation of the Middle Ear.*—This paper was a discussion of the subject in general, without any attempt to offer anything not found in the literature of the subject. The importance of a more full understanding of the importance and dangers of these affections by general practitioners was specially emphasized. He also reported a case of

*Double Optic Neuritis* from a violent fit of anger. The patient was a full-blooded, robust man, with a family history of insanity, and was subjected to more than usual excitement for a week previous to failure of vision, and the day previous he had a violent fit of anger. The ophthalmoscope showed the papillæ intensely engorged. Treatment: Free local blood-letting and large doses of chloral hydrate and potass. bromide. In three weeks from the time of beginning of failure of sight the neuritis had entirely disappeared. Vision, which when patient was first examined was right eye, fingers at ten feet, left eye $\frac{15}{100}$, had returned to normal condition, and so continues.

Dr. S. J. Jones made remarks commendatory of the report, insisting on the importance of a better understanding of these chronic ear troubles by practitioners.

Dr. F. H. Davis presented a volunteer paper on

*Inhalations*, in their application to diseases of the deeper pulmonary structures. Since reading a paper on the same subject, several months ago, before the Chicago Medical Society, he had made many new observations, and had added to the means of administering inhalations.

AFTERNOON SESSION.

The Committee on Nominations reported, recommending the following as officers for the ensuing year:

President—Dr. G. W. Jones, of Danville.

First Vice President—Dr. W. Hill, of Bloomington.

Second Vice President—Dr. G. W. Nesbitt, of Sycamore.

Treasurer—Dr. J. H. Hollister, of Chicago.

Assistant Secretary—Dr. W. T. Montgomery.

Committee of Arrangements for next meeting—Drs. J. Nevins Hyde, Norman Bridge, F. H. Davis, Roswell Park and E. F. Ingals. They recommended Chicago as the next place of meeting.

The report of the committee was accepted and adopted.

The Judicial Council reported through their Chairman, Dr. F. B. Hullar, recommending the expulsion from the Society of Dr. L. R. Williams, alias Lucas. The report was adopted unanimously.

Dr. Lucinda H. Corr, of Carlinville, from the Committee on Gynæcology, read an interesting paper on

*The Relative Value of Symptoms of Neurasthenia, as such, and the Remote Symptoms of Uterine Disease.*— She argued that while symptoms of neurasthenia might be due to general causes, uterine disease was capable of producing them, and frequently they were so produced. Neurasthenia in women was usually caused by uterine disease.

When symptoms of uterine disease and symptoms of neurasthnia were found in the same patient, it was generally true that the former had occurred first. There were three modes of treatment in vogue for such cases. The first was travel, hygiene, general tonics, etc. The next was local treatment of the uterine disorder. Of these two the latter was the best, as shown by results. The best treatment was a judicious combination of the two already mentioned.

Dr. Jewell remarked that the position assumed in the paper was correct.

Dr. J. H. Rauch next occupied the attention of the Society in a detailed statement of the doings of the State Board of Health since its organization. He also discussed the value, relations and means of execution of the Medical Practice act, and that for the gathering of vital statistics.

Dr. J. J. R. Patrick, of Belleville, read an essay on

*Medical Specialties*, but more especially the specialty of dental surgery in its intimate relation to the general practice of medicine.

*First.* Specialties necessary to human progress as it is impossible for one mind to acquire an accurate knowledge of the whole domain of medicine.

*Second.* The impossibility of drawing a line of demarcation between the different specialties and the general practice of medicine.

*Third.* The great abuse in constant practice by so-called dentists in using anæsthetics as an auxiliary in the extraction of teeth, in order to replace them with others less expensive.

*Fourth.* The many diseases of the mouth, which are constantly under the eye of the intelligent dentist, render him, if properly qualified, the proper person to treat such disesases, owing to his constant manipulation in that region.

*Fifth.* Showing that the mouth, being the vestibule of the alimentary canal, should receive more attention on the part of the general practitioner in the diseases of the body.

Dr. H. Z. Gill, a special committee on

*Croup*, read a lengthy paper in which he discussed the literature of the pathology of croup and diphtheria. He also discussed the treatment of these affections, and especially tracheotomy, and added to his former statistics of the operation in Illinois. The per cent. of recoveries from the operation up to date was 27½.

His conclusions as to the pathology of the two diseases were that there is nothing revealed by the microscopic or by the chemical examination of the deposit in the throat or elsewhere, by which the one disease may be distinguished from the other.

The next paper was by Dr. E. L. Harriott on

*Anæsthetics in Labor.* He had "circularized" the profession of the State and received replies from nearly twenty practitioners on the subject of his paper. All who had replied were pleased with the use of anæsthetics in labor, and most were warm in their enthusiasm. He advocated the use of anæsthesia much earlier than most. He would use it in the first stage of labor, and was sure it hastened the termination of this stage and of the completion of labor. He had never seen pains retarded by it; nor severe hæmorrhage. He would carry the agents to full insensibility, even in the first stage, and keep the patient under their influence most of the time till full delivery.

There was no hazard in this practice. No death had ever been reported from anæsthetics in labor.

Chloroform was his favorite agent and was the one used by most of his correspondents. This paper was discussed by Drs. Bird of Quincy, Palmer, McIntyre and others of St. Louis, and Reber of Shelbyville, and Dr. Steele.

A memorial was read from the Woman's Christian Temperance Union, asking the opinion of the society upon the necessity of ever using alcohol as a medicine, and whether ale and beer were beneficial as tonics.

It was, on motion, referred to a special committee of three.

The committee on Necrology presented their report, which included a biographical sketch of the late Dr. Thomas Bevan, which had been prepared by Dr. E. Ingals of the committee.

Dr. E. W. Lee, of Chicago, then read a paper on Tracheotomy.

This paper was discussed by Dr. Bird, who advocated operating without the tube.

Dr. Gill thought very much depended on the thorough care in the after treatment.

Dr. Porter, of St. Louis, advocated a longer incision both through the trachea and the overlying tissues. Nothing could be lost and much was gained in the ease with which the tube was removed and replaced and the trachea kept free from accumulations.

Dr. Gill introduced a resolution amending the by-laws so as to allow members to have copies of papers read before the society for publication in medical journals, provided it be with the explanation that they were read before the society. The resolution was passed.

THIRD DAY. MORNING SESSION.

The only scientific business was the reading of a paper by Drs. Lee and Fenger, of Chicago, on Tuberculosis of Joints, with three cases of extirpation and exhibition of two of the patients. As this paper is soon to appear in the JOURNAL AND EXAMINER we will not summarize it.

A resolution was introduced and passed to the effect that the advertising of quack medicines by newspapers was an imposition upon the people, to their pecuniary loss, and often to their physical injury and discomfort, and was unbecoming any publication making pretensions to teach morality and religion.

It was voted to instruct the secretary to send a copy of this resolution to each religious newspaper in the United States.

The special committee on the memorial from the Woman's Christian Temperance Union, presented a report, the purport of which was that while the society recommended temperance in all things, its members felt that alcohol was, in its place, a valuable medicine that could not be dispensed with.

The report was adopted, but a special committee was appointed to report on the subject more fully at the next meeting.

In appropriate resolutions the thanks of the society were tendered the local committee, the railroad companies, Profs. Hodges and Irwell for their evening lectures, and Judge Snyder for his able evening address. The society then adjourned.

---

